# Maternal mortality trends in Spain during the 2000-2018 period: the role of maternal origin

**DOI:** 10.1186/s12889-022-12686-z

**Published:** 2022-02-17

**Authors:** Santiago García-Tizón Larroca, Juan Arévalo-Serrano, Maria Ruiz Minaya, Pilar Paya Martinez, Ricardo Perez Fernandez Pacheco, Santiago Lizarraga Bonelli, Juan De Leon Luis

**Affiliations:** 1 Department of Obstetrics and Gynaecology, Hospital General Universitario Gregorio Marañón, Universidad Complutense de Madrid, O’donnell 48, 28029 Madrid, Spain; 2 Department of Internal Medicine, Hospital Universitario, Principe de Asturias, Universidad de Alcalá, de Alcalá de Henares, Madrid Spain

## Abstract

**Background:**

The available literature indicates that there are significant differences in maternal mortality according to maternal origin in high income countries. The aim of this study was to examine the trend in the maternal mortality rate and its most common causes in Spain in recent years and to analyse its relationship with maternal origin.

**Methods:**

This was a cross-sectional study of all live births as well as those resulting in maternal death in Spain during the period between 2000 and 2018. A descriptive analysis of the maternal mortality rate by cause, region of birth, maternal age, marital status, human development index and continent of maternal origin was performed. The risk of maternal death was calculated using univariate and multivariate logistic regression analyses, with adjustment for certain variables included in the descriptive analysis.

**Results:**

There was a total of 293 maternal deaths and 8,439,324 live births during the study period. The most common cause of maternal death was hypertensive disorders of pregnancy. The average maternal death rate was 3.47 per 100,000 live births. The risk of suffering from this complication was higher for immigrant women from less developed countries. The adjusted effect of maternal HDI score over maternal mortality was OR = 0.976; 95% CI 0.95 – 0.99; *p* = 0.048; therefore, a decrease of 0.01 in the maternal human development index score significantly increased the risk of this complication by 2.4%.

**Conclusions:**

The results of this study indicate that there are inequalities in maternal mortality according to maternal origin in Spain. The human development index of the country of maternal origin could be a useful tool when estimating the risk of this complication, taking into account the origin of the pregnant woman.

## Background

Maternal mortality is one of the most sensitive indicators of the quality of health care in each country [1]. This complication of pregnancy is catastrophic both for families and for society in general, constituting an important public and social health problem. This is aggravated in situations where certain factors such as economic, educational and legal inequality or the lack of opportunities for a specific group of women entail an increased risk of this pregnancy outcome [2].

Despite the decrease in the rate of maternal mortality in recent years, there is still a lack of significant progress in reducing the frequency of preventable causes of maternal death due to multiple and complex factors. This results in an estimate of approximately 300,000 maternal deaths per year worldwide due to pregnancy, childbirth and postpartum complications [3]. In 2015, the World Health Organization (WHO) released “Strategies towards ending preventable maternal mortality (EPMM)” (EPMM Strategies), a direction-setting report indicating global targets and strategies for reducing maternal mortality in the Sustainable Development Goal (SDG) period [4]. In this regard, several countries and international agencies have previously sought, through the Millennium Development Goals (MDGs), to improve the lives of people in more disadvantaged countries by globally reducing the rate of maternal death, among other objectives [5].

The monitoring of the progress of the MDG 5 when measuring the variations in the maternal death rate has shown that many countries lack quality data for the quantification of this complication. The UN’s Maternal Mortality Estimation Inter-Agency Group (consisting of the WHO, UNICEF, UNFPA, World Bank Group, and UNPD) has tried to produce reliable data on maternal mortality in each country since 1990 [6–8].

In this regard, the causes of maternal death are not always well recorded or identified. With data collected between 2003 and 2009 in 115 countries, Say et al. [9] were able to verify that approximately 70% of maternal mortality events were due to direct obstetric causes. However, the study data showed that these were incomplete and that the indirect causes were not well defined in up to one-fifth of the cases [9].

In addition to the causes of maternal death, there are certain socioeconomic and sociodemographic factors related to the lifestyle of women that can influence the outcome of their pregnancy. Maternal origin and immigration are particularly relevant.

Several authors have studied the influence of immigrant women on public health in destination countries, especially in Europe and North America. Obstetric outcomes appear to be worse in pregnant immigrants than in native women in Western countries. The published results are heterogeneous mainly due to the different ways of classifying immigrant women, either by race, country of origin or socioeconomic level of the place of origin [10–12]. Some authors have proposed using the human development index (HDI) of the country of maternal origin to classify pregnant women. This index collects very relevant information on the socioeconomic situation of the country of origin and its citizens, such as having a long and healthy life, acquiring knowledge and having a decent standard of living [13]. The HDI is prepared annually by the United Nations Development Program (UNDP), assigning a score of 0 to 1 and classifying each country into one of 4 groups: very high HDI, high HDI, medium HDI and low HDI. There are other sociodemographic indices that reflect the situation of development, progress and absence of inequality among citizens of the same country, such as the gender inequality index (GII). This index measures the lost human development in 3 important dimensions, i.e., reproductive health, political empowerment, and economic status, reflecting the distance required for a society to achieve full equality between women and men [14].

The correct classification of immigrant women is extremely important because their risk of severe morbidity and maternal mortality is increased when compared with that for the native population in developed countries [15].

The main objective of this study was to analyse the trend in the maternal mortality rate in Spain in recent years and to identify certain sociodemographic factors that could influence it, such as maternal origin, through different forms of classification.

## Methods

This was a cross-sectional study with data from all live births and the pregnancies that resulted in maternal death in Spain during the 2000-2018 period. The information was provided by the Spanish National Institute of Statistics (INE, for its abbreviation in Spanish) upon specific request by the authors. The INE approved this data to be published (reference number PB063/2021). Information was collected on the region of Spain where the delivery occurred and the year of delivery, maternal age, maternal marital status, continent of maternal origin, HDI of the country of maternal origin, GII of the country of maternal origin and the cause of maternal death classified based on the ICD-10.

First, the maternal mortality rate was calculated using the number of annual maternal deaths per 100,000 live births to assess trends during the study period. The definition of maternal death used in this study was that given by the WHO:

The death of a woman while she is pregnant or within 42 days after the termination of the pregnancy, regardless of the duration and site of pregnancy, due to any cause related to or aggravated by the pregnancy itself or its care, but not due to accidental or incidental causes [16].

In Spain, every maternal death must be reported to the INE through 3 documents: medical death certificate, which is filled out by the healthcare professional with the cause and the date of maternal death in accordance with the latest WHO recommendations, the statistical bulletin of judicial death and the statistical bulletin of childbirth.

A descriptive analysis of the sample was performed by calculating maternal mortality rates by population groups of pregnant women based on the cause of maternal death, the region of Spain where the birth occurred, the maternal age range, the maternal marital status, the maternal HDI group (1-very high, 2-high, 3-medium, and 4-low) and the continent of maternal origin (Europe, America, Africa, and Asia).

An initial comparative analysis was performed between 2 groups of pregnant women based on their belonging to more developed HDI groups (groups 1 and 2) and less developed HDI groups (groups 2 and 3) based on variables such as maternal age, maternal marital status, maternal GII and the most common causes of maternal death (haemorrhage, hypertensive disorders of pregnancy, infection and sepsis, amniotic fluid embolism, abortion and obstetric thromboembolism).

Linear regression analysis was carried out initially between the HDI variables of the country of maternal origin and the maternal mortality rate and between the GII of the country of maternal origin and the maternal death rate to assess the relationship between them.

Last, logistic regression analysis was performed using the following variables: continent of maternal origin, year in which birth occurred, maternal HDI, maternal HDI group and maternal GII. In addition, a new variable, HDI100, was created, resulting from multiplying the HDI score by 100. Indicator variables were used, with the category with the lowest maternal mortality serving as the reference. In this way, univariate analysis was performed with each of the variables, and multivariate analysis was performed with all variables.

The effect of maternal HDI score over maternal mortality was adjusted using backward estimative multivariate binary logistic regression with the predictors year in which birth occurred, immigrant status, continent of maternal origin and GII. Variable selection in the multivariate regression model was performed according to subject matter knowledge as predictors of maternal mortality. The order of selection to evaluate the inclusion or exclusion of the predictors was by descending statistical significance. The criteria taken to maintain or retire the predictors was the clinical significance, that is, the change of more or less than 10% of maternal HDI OR.

The results were expressed as odds ratios (ORs) with a 95% confidence interval, and *p* values < 0.05 were considered significant. All statistical analyses were carried out using STATA version 15.0 (Stata Corp, College Station, TX).

## Results

**Fig. 1:**
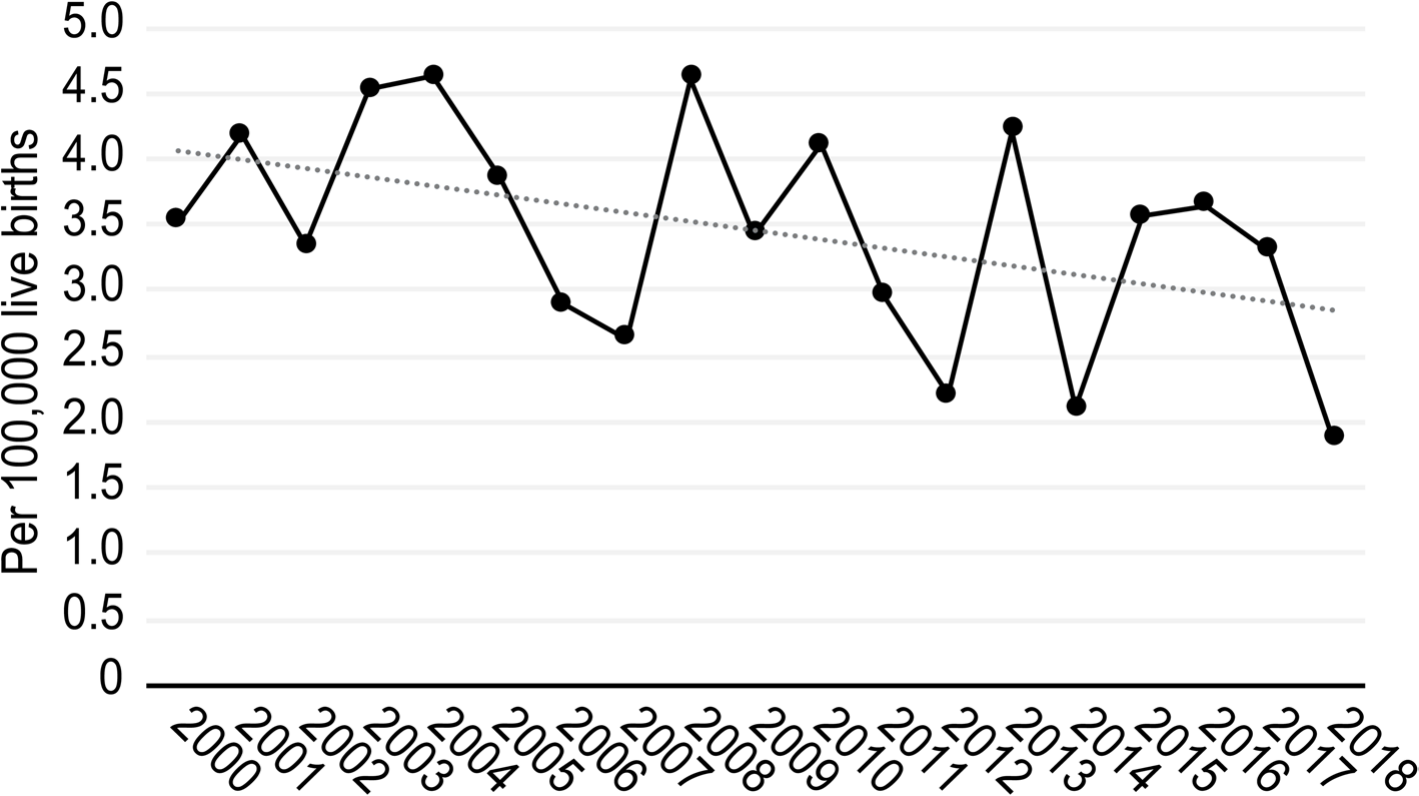
Maternal mortality rate trends in Spain during the 2000-2018 period

During the study period, data from 8,439,324 live births and 293 maternal deaths were collected; the data indicated that the average maternal mortality rate was 3.47 per 100,000 live births in Spain. Figure 1 shows a slightly decreasing trend in the rate of this complication year by year.

Table 1 provides the data on the most frequent causes of maternal death in Spain and the rates by region where delivery occurred. The most common cause of death in pregnant women was hypertensive disorders of pregnancy (19.4% of the total), followed by obstetric haemorrhage (18.7%). The region of Spain with the lowest rate of this complication was the Chartered Community of Navarra, located in the north of Spain, and the region with the highest was Melilla, with an average rate of 19.81 per 100,000 live births.

**Table 1:** Descriptive analysis of maternal mortality rate by ICD-10 cause, and region of delivery

Variables		Deaths, n (%)	Total live births (n)	Per 10^5^ live births (95% CI)
ICD-10 cause				
Ectopic pregnancy	O00	11 (3.75)	8,439,324	0.13 (0.05-0.2)
Pregnancy with abortive outcome (excluding ectopic pregnancy)	O01-O08	20 (6.82)	8,439,324	0.23 (0.13-0.34)
Oedema, proteinuria and hypertensive disorders of pregnancy, childbirth and the puerperium	O10-O16	57 (19.4)	8,439,324	0.67 (0.5-0.85)
Haemorrhage	O20 O44.1 O45 O46 O67 O72	55 (18.7)	8,439,324	0.65 (0.47-0.82)
Infection/sepsis	O75.2 O75.3 O85 O86 O41.1	21 (7.16)	8,439,324	0.24 (0.14-0.35)
Obstetric blood-clot embolism	O22.1 O22.3 O22.5 O22.8 O22.9 O87.0 O87.1 O.87.3 O87.8 O87.9 O88	23 (7.84)	8,439,324	0.27 (0.16-0.38)
Amniotic fluid embolism	O88.1	30 (10.2)	8,439,324	0.35 (0.22-0.48)
Complications of anaesthesia	O29 O74 O89	2 (0.6)	8,439,324	0.02 (0.00-0.05)
Rupture of uterus	O71.0 O71.2	9 (3)	8,439,324	0.1 (0.03-0.17)
Other direct causes		44 (15,01)	8,439,324	0.52 (0.36-0.67)
Indirect causes: Diseases of the circulatory system complicating pregnancy, childbirth and the puerperium	099.4	4 (1.3)	8,439,324	0.04 (0.00-0.09)
Diseases of the circulatory system complicating pregnancy, childbirth and the puerperium	O98, O99.1-3, 5-9	7 (2.3)	8,439,324	0.08 (0.02-0.39)
Obstetric death of unspecified cause	O95	10 (3.4)	8,439,324	0.11 (0.04-0.19)
Region				
Andalusia		88 (30)	1,642,208	5.35 (4.23-6.47)
Aragon		12 (4.1)	220,491	5.44 (2.36-8.52)
Balearic Islands		9 (3.1)	206,731	4.35 (1.51-7.2)
Catalonia		46 (15.7)	1,427,482	3.22 (2.3-4.15)
Canary Islands		14 (4.77)	342,649	4.08 (1.94-6.22)
Cantabria		3 (1)	91,215	3.28 (0.43-7.01)
Castilla La Mancha		8 (2.73)	346,581	2.3 (0.71-3.9)
Castile and Leon		12 (4.1)	351,917	3.41 (1.48-5.34)
Community of Madrid		28 (9.55)	1,292,946	2.16 (1.36-2.96)
Chartered Community of Navarra		1 (0.34)	118,432	0.84 (0.08-2.49)
Valencian Community		27 (9.21)	892,936	3.02 (1.88-4.16)
Extremadura		4 (1.36)	183,018	2.18 (0.04-4.32)
Galicia		7 (2.38)	388,203	1.8 (0.46-3.13)
Basque Country		6 (2.04)	370,431	1.62 (0.32-2.91)
Principality of Asturias		10 (3.41)	155,008	6.45 (2.45-10.44)
Region of Murcia		7 (2.38)	315,854	2.21 (0.57-3.85)
La Rioja		3 (1.02)	54,755	5.47 (0.72-11.6)
Ceuta		2 (0.68)	26,393	7.57 (2.92-18.1)
Melilla		6 (2.04)	30,283	19.81 (3.96-35.6)

Table 2 provides data on maternal death rates by age group, maternal marital status, HDI group of maternal origin and continent of maternal origin; pregnant women aged 40 years or older, married women, those belonging to the least developed HDI group (group 4), with a rate of 11.4 per 100,000 live births, and patients of Asian origin had the highest rates of maternal death, respectively. Overall, pregnant immigrants experienced higher rates of maternal death than did native women.

**Table 2:** Descriptive analysis of maternal mortality rate by maternal age, marital status, HDI group, maternal continent of origin and maternal origin

Variables	Deaths, n (%)	Total live births (n)	Per 10^5^ live births (95% CI)
Maternal Age			
≤ 20	5 (1.7)	210,655	2.37 (0.3-4.45)
20 - 29	60 (20.47)	2,603,323	2.3 (1.72-2.88)
30 – 39	183 (94.8)	5,185,060	3.52 (3.01-4.04)
≥ 40	45 (15.35)	440,286	10.22 (7.23-13.2)
Marital status			
Married	208 (71)	5,611,091	3.7 (3.2-4.21)
Not married	85 (29)	2,828,233	3 (2.36-3.64)
HDI group			
1-Very high	236 (80.54)	7,286,687	3.23 (2.82-3.65)
2-High	33 (11.26)	634,656	5.2 (3.42-6.97)
3-Medium	15 (5.2)	439,037	3.41 (1.68-5.14)
4-Low	9 (3.1)	78,944	11.4 (3.95-18.84)
Maternal continent of origin			
	234 (79.86)	7,236,218	3.23 (2.81-3.64)
America	26 (8.87)	622,458	4.17 (2.57-5.78)
Africa	26 (8.87)	469,946	5.53 (3.4-7.65)
Asia	7 (2.3)	111,702	6.26 (1.6-10.9)
Maternal origin			
Native	225 (76,8)	6,819,919	3.29 (2.86-3.73)
Immigrant	68 (23.2)	1,619,405	4.2 (3.2-5.19)

Table 3 shows the comparison of cases of maternal death between groups with a higher degree of human development (groups 1 and 2) and groups with a lower HDI (groups 3 and 4) in terms of maternal characteristics and more common causes of maternal death. The only statistically significant differences were the GII for each group.

**Table 3:** Comparison of cases of maternal death between groups with a higher degree of human development (groups 1 and 2) and groups with a lower HDI (groups 3 and 4)

Variable	HDI 1-2	HDI 3-4	p value
Maternal age, mean (SD)	33.8 (5.76)	32.2 (5.58)	0.19
Not married, n (%)	82 (30.4)	3 (12.5)	0.06
GII, mean (SD)	0.11 (0.1)	0.49 (0.05)	< 0.001
Cause of maternal death:			
Haemorrhage, n (%)	56 (20.81)	7 (41.2)	0.34
Hypertensive disorders, n (%)	51 (18.9)	6 (25)	0.54
Infection/sepsis, n (%)	20 (7.43)	1 (4.16)	0.55
Amniotic fluid embolism, n (%)	28 (10.4)	1 (4.16)	0.32
Abortive outcome, n (%)	19 (7.06)	1 (4.16)	0.58
Obstetric blood-clot embolism, n (%)	23 (8.55)	1 (4.16)	0.43

**Fig. 2:**
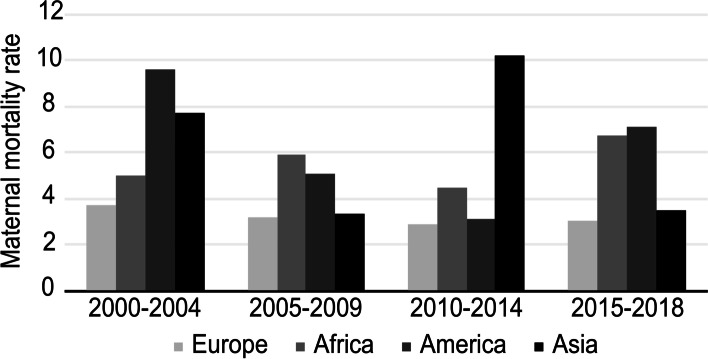
Maternal mortality rate by continent of maternal origin and period

**Fig. 3:**
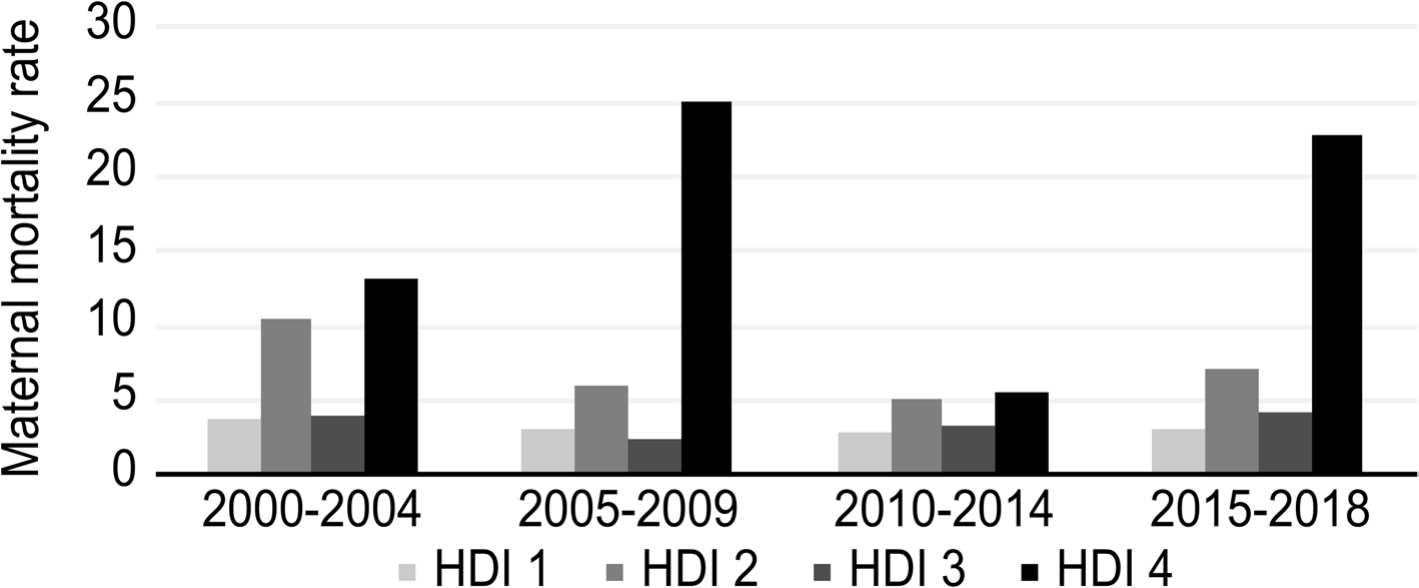
Maternal mortality rate by maternal HDI group of maternal origin and period

Figures 2 and 3 show, respectively, the trend over time in the rate of maternal death by continent of maternal origin and HDI group of maternal origin. Compared to the rest of the groups, European pregnant women had the lowest rates of maternal death in all time periods studied, and women belonging to HDI groups 2 and 4 showed the highest rates in the same time intervals.

**Fig. 4:**
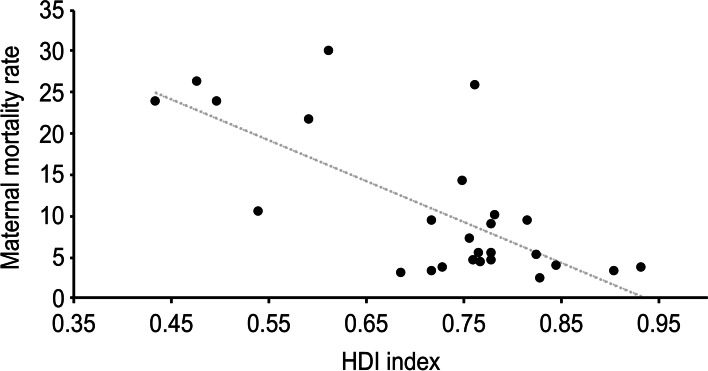
Relationship between the HDI of the country of maternal origin and maternal death rate

**Fig. 5:**
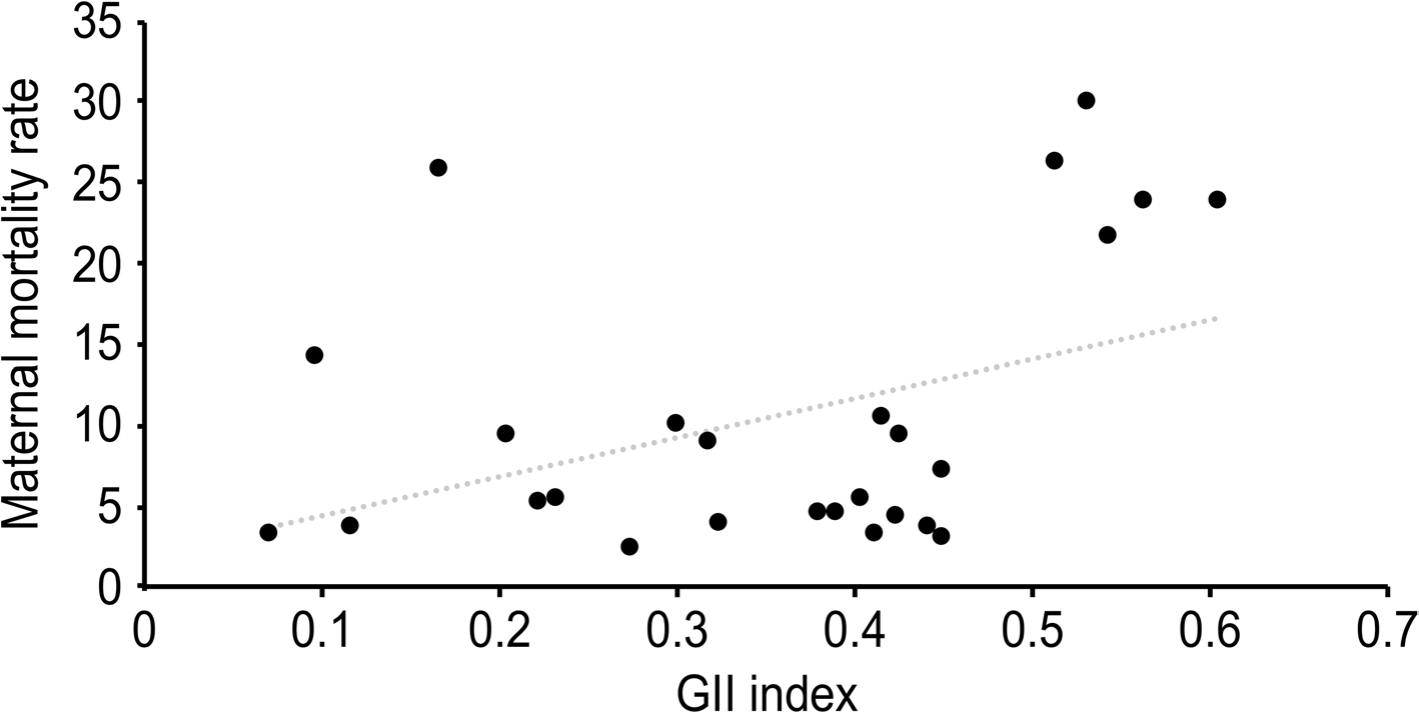
Relationship between the GII of the country of maternal origin and maternal death rate

Figure 4 shows the statistically significant relationship, based on linear regression analysis, between the HDI of the country of maternal origin and the maternal death rate (y = − 49.447x + 46.329, R^2^ = 0.5149, *p* < 0.01), such that the lower was the HDI of maternal origin, the higher was the maternal mortality rate. Figure 5 shows the same type of regression analysis between the GII of the country of maternal origin and the maternal mortality rate (y = − 49.447x + 46.329, R^2^ = 0.5149, *p* = 0.038); the lower was the GII of maternal origin, the lower was the maternal mortality rate.

Table 4 provides the results of the final univariate logistic regression analysis taking into account the HDI100 as a variable of interest and the HDI group, GII, year of delivery and continent of maternal origin as potential confounders in the initial maximal model. The model adjusted for these predictor variables indicated that for each 0.01-point decrease in the HDI of maternal origin, the risk of maternal death significantly increased by 2.4%.

**Table 4:** Final univariate logistic regression analysis

	Univariate		
	p	OR	CI 95%
*Nationality*			
Non-spanish	0.386	1.27	0.74 – 2.20
Spanish^a^		1	
*Continent*	0.025		
Europe^a^		1	
Africa	0.132	1.71	0.85 – 3.44
America	0.411	1.29	0.70 – 2.38
Asia	0.289	1.68	0.65 – 4.37
Other	0.002	26.49	3.34 – 210.02
*Year*	1.00		
2000^a^		1	
2001	0.899	1.19	0.08 – 17.12
2002	0.969	0.95	0.07 – 13.40
2003	0.850	1.29	0.09 – 17.49
2004	0.837	1.31	0.10 – 17.47
2005	0.944	1.10	0.08 – 14.44
2006	0.882	0.82	0.06 – 10.79
2007	0.823	0.75	0.06 – 9.36
2008	0.829	1.31	0.11 – 15.45
2009	0.984	0.98	0.08 – 11.62
2010	0.902	1.17	0.10 – 13.80
2011	0.893	0.84	0.07 – 10.22
2012	0.714	0.63	0.05 – 7.75
2013	0.885	1.20	0.10 – 14.45
2014	0.690	0.60	0.05 – 7.51
2015	0.991	1.01	0.08 – 12.26
2016	0.977	1.04	0.09 – 12.43
2017	0.960	0.94	0.08 – 11.22
2018	0.624	0.53	0.04 – 6.59
*GII score × 100*	0.172	1.01	0.99 – 1.03
*HDI group*	0.001		
Group 1^a^	1		
Group 2	0.110	1.61	0.90 – 2.87
Group 3	0.894	1.06	0.48 – 2.31
Group 4	< 0.001	5.07	2.28 – 11.53
***HDI score × 100***	**0.048**	**0.976**	**0.95 – 0.99**

^a^Reference

The result of estimative multivariate logistic regression analysis taking into account the HDI score × 100 as a variable of interest and the immigrant state, GII, year of delivery and continent of maternal origin as potential confounders in the initial maximal model was OR = 0.976; 95% CI 0.95 – 0.99; *p* = 0.048. The model adjusted for these predictor variables indicated that for each 0.01-point decrease in the HDI of maternal origin, the risk of maternal death significantly increased by 2.4%.

## Discussion

To our knowledge, this is the most up-to-date study on maternal mortality data in Spain. Although this maternal complication of pregnancy is recorded through the INE, there are no periodic analyses by health institutions that systematically reflect the trend or characteristics of this event in Spain. In this regard, the WHO stated that nations should maximize their efforts in strengthening health systems to collect high-quality data to respond to the needs and priorities of women and girls and ensure accountability to improve the quality of care and equity [17].

The maternal mortality rate in Spain is one of the lowest observed in countries in our region, with slight fluctuations year by year, which reflects, in part, the good health care provided for pregnancy, childbirth and postpartum complications and the universal access of the population to this health care. Other countries with similar or even higher levels of development, such as Norway and Canada, show maternal mortality rates in recent years between 5.1 and 12 deaths per 100,000 live births [18, 19]. The increase in the rate of this complication in these countries, such as Canada, was proposed to be the result of improvements in vital statistics registration data and due to the switch from the ICD9 to ICD10 when classifying this complication. In this regard, we do not know if maternal deaths are correctly quantified in Spain, although some authors claim that there may be inadequate identification and recording of maternal deaths in up to 40% of cases, which would reflect a clear underestimation of maternal mortality [20].

One of the most important findings of our research was the identification of the most common causes of maternal death in Spain. The most prevalent causes were hypertensive disorders of pregnancy, closely followed by obstetric haemorrhage. Other causes, in order of frequency, were other direct obstetric causes in up to 15% of the cases, amniotic fluid embolism in 10% of cases, infection and sepsis and obstetric thromboembolism; these data allows recognition of the problems that require greater optimization with respect to the allocation of health resources most necessary in Spain. These data do not differ much from those previously published by Fernandez et al. [21], who reported that the most prominent causes of maternal death in Spain were hypertensive disorders of pregnancy and postpartum complications in 22.6 and 23.3% of cases, respectively.

A descriptive analysis of the causes and trends of maternal mortality in Spain during the period between 1999 and 2015 also indicated that the most common causes responsible for this perinatal event were, in order of frequency, obstetric haemorrhage and hypertensive disorders of pregnancy [22]. The most relevant research on maternal mortality at the international level was published in 2015, in which a global and regional review of data from 186 countries during the 1990–2015 period identified the 8 main causes of maternal death. The results of the study indicated that, overall, obstetric haemorrhage is the most frequent cause of maternal death in most countries and that it is potentially avoidable with adequate obstetric management as well as the use of appropriate health resources [23].

Our results showed differences in the rate of this complication among the different regions of Spain where delivery occurred. The Chartered Community of Navarra and the Basque Country had the lowest rates while Melilla and Ceuta had the highest rates in the national territory. We do not know the underlying reasons for this situation, although possible factors include greater immigration from Africa in southern regions of Spain and each region having its own health system independent of the rest of the national territory. The distinguishing characteristics of different populations, the influx of immigrants with certain profiles and the inequality in the health benefits of each nation could justify the existence of health inequities in general and in reproductive health specifically among citizens of European countries, as was observed in this study [24, 25].

Maternal age could play an important role in the risk of maternal death, although its influence on this perinatal outcome was not specifically analysed. The group of women aged 40 years or older had a crude risk on the order of 3 to 5 times higher than that observed for women in other age groups in Spain during the study period. The risk of adverse perinatal events and complications during pregnancy is significantly increased with maternal ages greater than 40 years [26, 27]. Sheen et al. [28] pointed out that women in the age group of 45 years or older were those who had a greater risk of caesarean delivery, preeclampsia, postpartum haemorrhage, gestational diabetes, puerperal thrombosis and hysterectomy as severe complications of pregnancy.

When observing maternal death rates by maternal origin, the results of this study revealed very relevant findings regarding its influence on this pregnancy complication. First, the rates of maternal death were higher in the HDI groups comprising less developed countries, especially group 4 (very low HDI), with a crude risk 3 to 4 times higher than that for group 1. Regarding the continent of origin, with respect to European pregnant women, the rest of the women had higher rates of maternal death, more markedly for those whose continents of origin were Asia and Africa. This finding was already suspected due to previous publications in which maternal death and severe acute maternal morbidity events occurred more frequently in foreign women from less developed countries [13, 29].

Through linear regression analysis, we were able to verify that the lower the HDI and the higher the GII of the country of maternal origin, the higher was the maternal death rate, revealing how important it is to conduct further research on these aspects of development by classifying the origin of immigrant women in our country. In addition, when performing multivariate regression analysis adjusted for different covariates, we observed that a decrease of 0.01 points in the maternal HDI score generates a significant increase in the risk of maternal death. This allows a more accurate calculation of the added risk that a patient has of dying from pregnancy complications as a function of variations in this variable.

The HDI of the country of maternal origin simplifies and captures very important sociodemographic and economic characteristics regarding the development of each nation and provides a quantitative dimension. This could explain why its use may be valuable when analysing the specific risk of immigrant pregnant women suffering certain complications of pregnancy in developed countries, such as Spain, because maternal origin and various social determinants, such as family income, education level, degree of social exclusion and adequate access to emergency health services and pregnancy monitoring, have a very influential role in pregnancy outcomes [15, 30, 31].

Regarding the limitations of this study, we recognize that there are several. First, as previously mentioned, it is unknown whether all maternal deaths were correctly reported to the INE during the study period in Spain and whether this issue would result in an underestimation of the rates of this complication. Furthermore, for unclear reasons, there was also a nonnegligible percentage of maternal mortality cases with unspecified causes. As unspecified cases were very infrequent, it is possible that there would not be differences in terms of the characteristics of the pregnant women and the causes of maternal death when comparing higher HDI groups with lower HDI groups. In addition, in the multivariate analysis, the fact that there were relationships that were not significant can be explained by the fact that there were very few deaths with respect to the large number of births without mortality; therefore, the proportion of maternal deaths in all the groups analysed was very small, and it was difficult to find significant differences. Another limitation of our multivariate analysis model was the lack of adjustment for relevant variables, such as maternal age, body mass index, type of health care centre or pre-existing maternal conditions that are of interest in the study of maternal mortality.

Last, cases that fell within the definition of late maternal death and those that corresponded to the death of a woman from direct or indirect obstetric causes more than 42 days but less than one year after termination of pregnancy were not included [32]. These cases are equally important due to the very serious social connotations and the severe impact that the death of the mother produces on families.

## Conclusions

The maternal mortality rate in Spain is one of the lowest in the world, although it is possible that it is necessary to improve data collection systems when reporting this event as well as to periodically analyse its causes and most frequent risk factors in Spain. Maternal mortality occurs more frequently, with a significantly increased risk, in immigrant women from underdeveloped countries. This is why the use of maternal origin classification systems such as the HDI score may more precisely profile the risk of maternal death in pregnant women. This index includes extremely important aspects related to the characterization of the sociodemographic profile of patients.

## Data Availability

Any additional information regarding the study will be shared upon request by the corresponding author.

## Abbreviations

EPMM: Ending preventable maternal mortality
GBD: Global Burden of Disease
UN: United Nations
UNICEF: United Nations Children’s Fund
UNFPA: United Nations Fund For Population Activities
UNPD: United Nations Procurement Division
HDI: Human development index
GII: Gender Inequality Index
INE: Instituto Nacional de Estadistica
SDG: Sustainable Development Goal
USA: United States of America
WHO: World Health Organization
ICD: International Classification of Diseases
